# Revertant Fibers in the *mdx* Murine Model of Duchenne Muscular Dystrophy: An Age- and Muscle-Related Reappraisal

**DOI:** 10.1371/journal.pone.0072147

**Published:** 2013-08-28

**Authors:** Sarah R. Pigozzo, Lorena Da Re, Chiara Romualdi, Pietro G. Mazzara, Eva Galletta, Sue Fletcher, Stephen D. Wilton, Libero Vitiello

**Affiliations:** 1 Centre for Neuromuscular and Neurological Disorders, University of Western Australia Queen Elizabeth II Medical Centre, Nedlands Western Australia, Australia; 2 Department of Biology, University of Padova, Padova, Italy; New Jersey Medical School, University of Medicine and Dentistry of New Jersey, United States of America

## Abstract

Muscles in Duchenne dystrophy patients are characterized by the absence of dystrophin, yet transverse sections show a small percentage of fibers (termed “revertant fibers”) positive for dystrophin expression. This phenomenon, whose biological bases have not been fully elucidated, is present also in the murine and canine models of DMD and can confound the evaluation of therapeutic approaches. We analyzed 11 different muscles in a cohort of 40 *mdx* mice, the most commonly model used in pre-clinical studies, belonging to four age groups; such number of animals allowed us to perform solid ANOVA statistical analysis. We assessed the average number of dystrophin-positive fibers, both absolute and normalized for muscle size, and the correlation between their formation and the ageing process. Our results indicate that various muscles develop different numbers of revertant fibers, with different time trends; besides, they suggest that the biological mechanism(s) behind dystrophin re-expression might not be limited to the early development phases but could actually continue during adulthood. Importantly, such finding was seen also in cardiac muscle, a fact that does not fit into the current hypothesis of the clonal origin of “revertant” myonuclei from satellite cells. This work represents the largest, statistically significant analysis of revertant fibers in *mdx* mice so far, which can now be used as a reference point for improving the evaluation of therapeutic approaches for DMD. At the same time, it provides new clues about the formation of revertant fibers/cardiomyocytes in dystrophic skeletal and cardiac muscle.

## Introduction

Duchenne muscular dystrophy (DMD, OMIM #310200) is the most common X-linked recessive disease, with an incidence of approximately 1 every 3500 newborn males. In the vast majority of cases, DMD is caused by protein truncating mutations that either disrupt the reading frame or cause premature termination of translation of the dystrophin-encoding gene, which in turn lead in the lack of functional protein. Even in the presence of frame-shifting deletions of tens to hundreds of kilobases within the dystrophin gene, however, occasional dystrophin-positive “revertant fibers” (RFs) can be found in about 50% of DMD patients, in whom they can account for up to 7% of the total [1]–[9]. The same phenomenon is found also in murine and canine models for DMD: the mdx mouse [10], [11] and GRMD (Golden Retriever Muscular Dystrophy) [12] and cxmd dogs (Canine X-linked Muscular Dystrophy) [13]. Dystrophin re-expression occurs not only in skeletal but also in cardiac muscle, albeit only at a much lower frequency [14].

The presence of revertant fibers has no effect on the clinical phenotype; dystrophin expression is limited to few nuclear domains, with a longitudinal extension spanning 100 to 300 µm (but occasionally up to 900 µm) [6], and such “patchy” distribution cannot protect RFs from sarcolemmal damage [6], [9].

At present, the biological mechanism(s) of formation of revertant fibers are still poorly known. It has been reported that in newborn mice RFs appear as short segments of sporadic single fibers, which then expand over time to form clusters of dystrophin positive fibers [15]. The number of RFs in *mdx* skeletal muscle then increases with age [16] and the same finding has been shown in human DMD muscles [4], [6]. These data have suggested a clonal model for their appearance and expansion [5], [16], [17], according to which reversion would occur in a few myogenic progenitors at an early stage of muscle development and such revertant precursors would then give rise to the proportionally small number of dystrophin-positive fibers that can be seen in newborn mice. The expansion of the revertant precursors during the processes of muscle degeneration/regeneration that characterize dystrophic muscle, coupled to a better resistance of revertant areas to degeneration, would then lead to the increase in the number of RFs in time [18]. However, these conclusions derive from studies that analyzed just few limb muscles in small numbers of animals and no systematic studies with time course analyses have been reported for the heart.

Here we show the results of the largest study on RFs in *mdx* mice reported so far, in which we considered four different age groups: 2, 6, 12 and >18 months. In our experience, the number of revertant fibers in *mdx* muscles always showed a wide variability, both between different mice and between different muscles of the same mouse. For this reason, a group size of ten animals was chosen in order to be able to perform statistical analyses, which were carried on a total of 11 different muscles from three distinct groups: locomotor, respiratory (primary and accessory) and cardiac. More specifically, we considered 7 locomotor muscles (tibialis anterior, extensor digitorum longus, plantaris, soleus, quadriceps femoris, gastrocnemius, triceps brachii), 3 respiratory muscles (diaphragm, intercostal, pectoralis major) and the heart (ventricles).

We chose to perform repeated measures ANOVA tests, instead of the t-Student approach that has been applied so far in most studies about RFs, as we deemed that analysis of variance was a more suitable approach for a multi-level analysis, especially in a complex model that does not consider just comparisons between data pairs but deals with multiple factors and their interactions.

Our results support the hypothesis of a clonal expansion of revertant fibers starting from a small pool of muscle progenitor, and provide a reliable reference for the percentage of endogenous revertant fibers in the *mdx* mice when assessing the efficiency of therapeutic approaches aimed at restoring dystrophin expression. Interestingly, they also suggest the possibility that de-novo reversion phenomena could also occur during the whole lifespan of the animals and that such progression could also take place in the myocardium.

## Materials and Methods

### Animals

The colony had been previously established using breeding pairs obtained from Jackson Laboratories, strain C57BL/10ScSn-*Dmd^mdx^*. Animals had *ad libitum* access to food and water in standard cages containing plastic pipe sections for shelter and environment enrichment.

For the present study we used a total of 40 male mice, divided into four age groups; group 1: two month-old animals; group 2: six month-old animals; group 3∶12 month-old animals; group 4: animals older than 18 months. Each time point had a tolerance of ±5%. At sacrifice, the following muscles were harvested and snap-frozen in liquid nitrogen-cooled isopentane: tibialis anterior (TA, whole), extensor digitorum longus (EDL, whole), plantaris (PL, whole), soleus (S, whole), quadriceps femoris (Q, whole), gastrocnemius (GC, whole), triceps brachii (TRIC, whole), pectoralis major (PT, proximal half), intercostal (IC, between 7^th^ and 10^th^ ribs, sternal region), diaphragm (D, whole) and heart (H, ventricles).

### Ethic Statement

The animals used for this work were bred and handled in the animal facility of the Vallisneri Biology Building of the University of Padova, following the relevant national bylaws (“D.L. 27-1-92, numero 116, circolare applicativa del Ministero della Sanità numero 8 del 22-4-94”). The project was carried out with the authorization of the University of Padova Ethic Committee for Animal Experimentation.

### Immunohistochemistry and Collection of Raw Data

Muscles were sectioned using a cryotome; each sample was cut in two at the middle and one half was transversally sectioned, obtaining from ten to twelve 9 µm sections, ∼120 µm apart. For immunostaining, slides were first fixed in paraformaldehyde (2% in PBS buffer), saturated in 10% horse serum in PBS and incubated (one hour at room temperature) with an anti dystrophin polyclonal antibody (raised in rabbit, GeneTex 15277) diluted 1∶100 in PBS containing 1%BSA. Secondary antibody was a Cy3-conjugated anti rabbit, diluted 1∶200 and also incubated for one hour at room temperature. After staining, slides were mounted in Fluoroshield-Dapi Mounting Medium (Sigma). Examples of muscle sections containing different numbers of revertant fibers are shown in Figure S1.

Slides were analyzed using a Leica 5000 microscope, equipped with the LAS software. Each muscle section was visually analyzed in its entirety by the same operator and the desired data (total number of RF, number of isolated RF, number and size of RF clusters) were recorded. Fibers were counted as revertant only when the whole membrane circumference was stained in cross-sections. According to what previously reported [18], RFs adjacent to each other were considered as a single cluster. The area of each section was determined from composite images using the Photoshop CS software.

### Statistical Analysis

Repeated measures mixed effects analysis of variance was preliminarily performed in order to evaluate the significant factors to be included in the final model. Differences between muscle types and age groups were found to be significant, whereas mice from the same group and different sections from the same muscle were not.

Repeated measures mixed effects analysis of variance was then carried out by running single trait linear models [19], considering the number and spatial organization of the fibers as variable traits. Measures of the same subject have been modeled with a random effect while the type of muscle (TA, EDL, SOL, PL, GC, Q, TRIC, PT, IC, D and H; 11 levels), the group to which mice belonged (groups 1–4, 4 levels) and their interaction were considered as fixed effects. Statistical significance was set at P<0.05.

As RFs often extend for a length of several hundreds microns, the same fiber could appear in multiple sections of the same muscle, especially in older animals and in large clusters; therefore, a correct statistical model had to take into account the dependence structure existing between the various sections of each muscle.

## Results

### The Number of Revertant Fibers Varies According to Muscle Type and Animals’ Age

Initially, we performed a preliminary repeated measures analysis of variance on our dataset, described in details in material and methods, showing that both muscle type and animal age were significant parameters.

**Figure 1:**
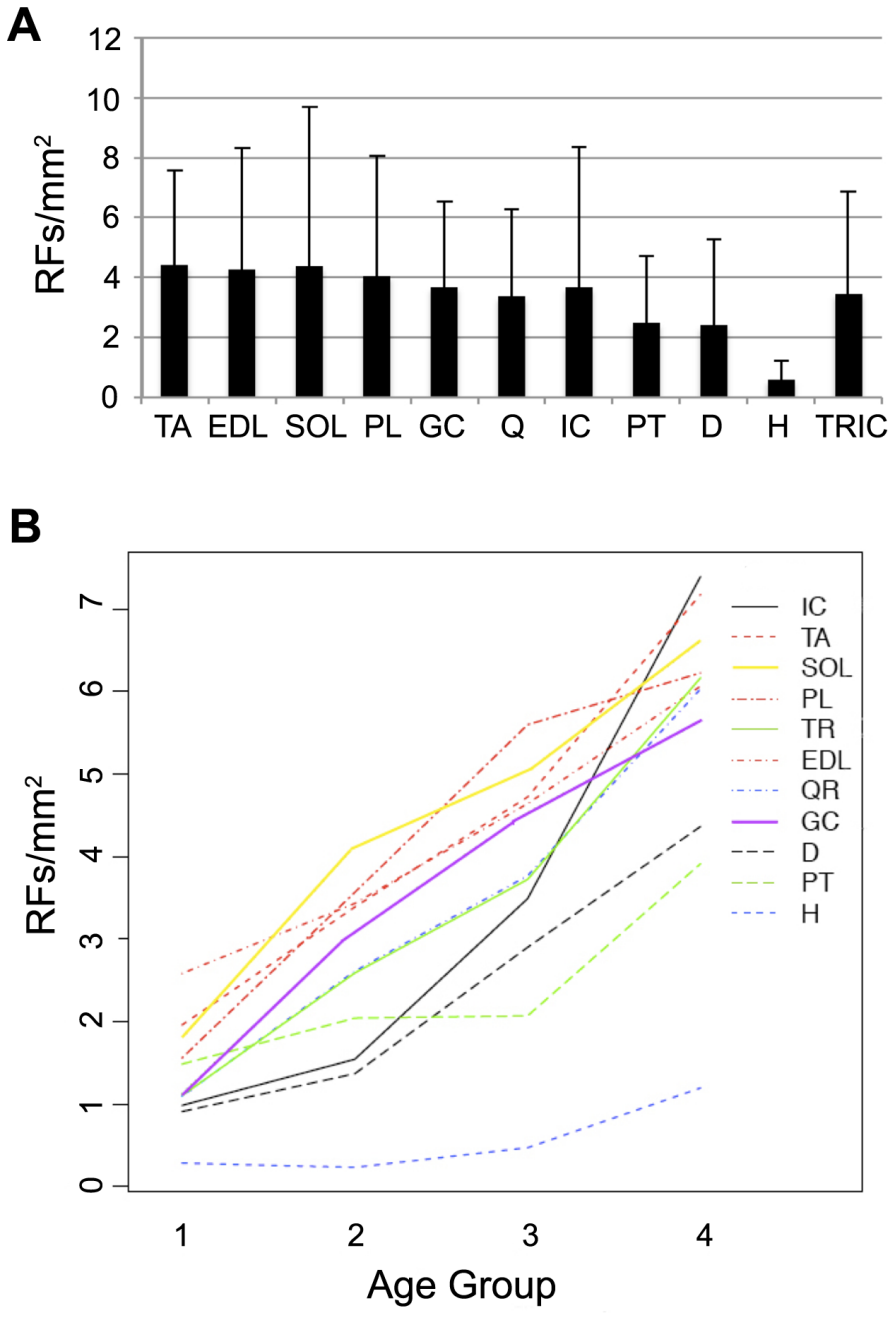
Number of revertant fibers (or of cardiomyocytes, in the case of heart) per square millimeter in different muscles. (A) Values calculated without differentiating between age groups; numbers were obtained by averaging the single RF/mm^2^ value of each of the 10–12 sections per muscle, for all forty mice. Abbreviations are as follows: TA, tibialis anterior; EDL, extensor digitorum longus; Q, quadriceps; SOL, soleus; GC, gastrocnemius; PL, plantaris; D, diaphragm; H, heart; IC intercostal; PT, pectoralis; TRIC, triceps. (B) Interaction between the number of revertant fibers per square millimeter (Y-axis) and the age of the animals (X-axis). For each muscle, the segmented line joins the four values of RFs/mm^2^, one for each age group; data were obtained by averaging the counts of all the sections, 10–12 per muscle, for ten animals. Note that the order of the muscles’ names at the right side of the chart reflects the order of the values found for group 4.

We then proceeded to compare the number of RFs per mm^2^ between different muscles, considering the average value between all age groups (Figure 1A and Table 1); this showed that diaphragm and pectoralis had fewer RFs than all other skeletal muscles (2.4 and 2.5/mm^2^, respectively) and the difference was highly significant (p<0.001). At the other end of the spectrum, tibialis anterior and soleus had the highest number of RFs (4.4/mm^2^) and the difference was statistically significant with all other muscles (p<0.05 or lower), with the exception of EDL and plantaris. Cardiac muscle had the lowest number of revertant cardiomyocytes, 0.6/mm^2^ (please note that in the charts dystrophin-positive cardiomyocytes are indicated as RFs).

**Table 1:** Average (± stdev) of RFs/mm^2^ in different muscles, at each age group.

	GROUP 1	GROUP 2	GROUP 3	GROUP 4
TA	1.96±2.04	3.39±1.98	4.72±2.17	7.17±3.45
EDL	2.58±3.49	3.35±2.75	4.6±3.01	6.06±5.38
SOL	1.8±2.28	4.13±3.25	5.03±5.78	6.61±7.35
GC	1.13±0.96	3.12±1.89	4.45±2.28	5.63±3.28
PL	1.56±1.8	3.57±3.74	5.6±4.14	6.22±4.43
Q	1.09±0.54	2.61±1.95	3.77±2.02	6.03±3.82
TRIC	1.09±1.75	2.59±1.35	3.72±2.23	6.19±4.61
PT	1.48±2.09	2.02±2.36	2.07±1.71	3.91±2.07
D	0.91±0.89	1.37±2.15	2,9±2.28	4.36±3.86
IC	0.98±0.84	1.54±1.26	2.96±3.71	7.39±6.22
H	0,28±0.50	0.23±0.27	0.47±0.51	1.19±0.66

We then analyzed how the number of RFs varied in the different muscles, as a function of animals’ age. As shown in Figure 1B, all skeletal muscles exhibit an upward trend, whose significance was confirmed in all instances by statistic analysis (p<0.001) when comparing the data for age group 1 and group 4. Differences were also always significant when comparing age groups 1–3 and groups 2–4, with p<0.001 (except for EDL between groups 1–3, in which p<0.1), whereas p values were almost invariably >0.05 when comparing sequential groups (i.e., 1–2, 2–3 and 3–4).

Heart showed a different situation, with a total number of revertant cardiomyocytes that remained consistently lower than what seen for RFs in skeletal muscles at all time points. An increasing trend in the number of revertant cardiomyocytes per mm^2^ in connection with age was still present, but the differences did not reach statistical significance.

### Distribution of RFs in Muscle Sections

**Figure 2:**
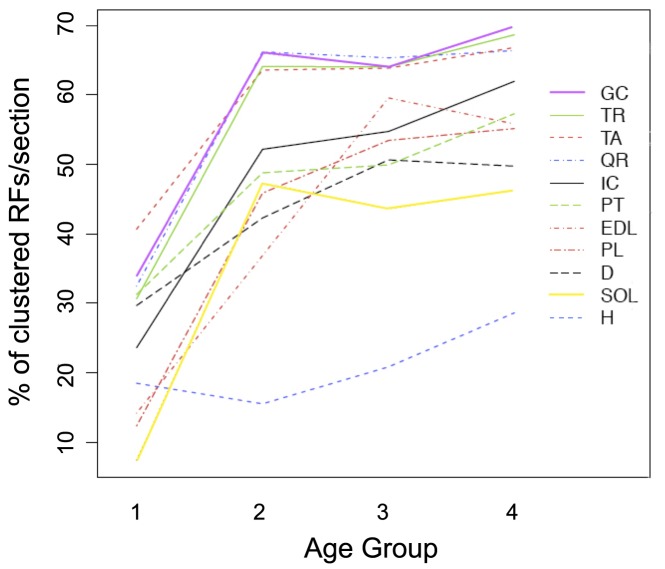
Interaction between the percentage of clustered revertant fibers per section and the age of the animals. Chart is organized as in Figure 1B; for each muscle, values were obtained by averaging the percentage of clustered fibers over the total for each section.

Next, we studied the spatial organization of RFs in dystrophic muscles, differentiating between isolated or clustered fibers. For this purpose, we analyzed the percentage of clustered RFs over the total RF number for each muscle in each age group. As shown in Figure 2, skeletal muscles, with the exception diaphragm and pectoralis, displayed a highly significant increase between age groups 1 and 2 (p<0,001), followed by stabilization to a plateau. Once again, all muscles showed a significant difference between group 1 and 4 (p<0.05). Interestingly, when taking into account all four time points, the largest limb muscles: TA, GC, Q and TRIC, showed significantly higher percentages of clustered RFs compared to all other muscles (p<0.05 or lower). As for the cardiac muscle, it showed an increasing trend with age, but the differences between groups did not reach statistical significance.

In order to investigate the possible presence of newly formed RFs (i.e., derived from satellite cells that acquired the capability of producing functional dystrophin during adulthood) we assessed the number of single revertant fibers that were separated from a neighboring RF by at least 2 fibers. The rational of this choice was to minimize the possibility that they could derive from a previously existing cluster in which some revertant fibers had died and had been replaced by non-revertant ones.

**Figure 3:**
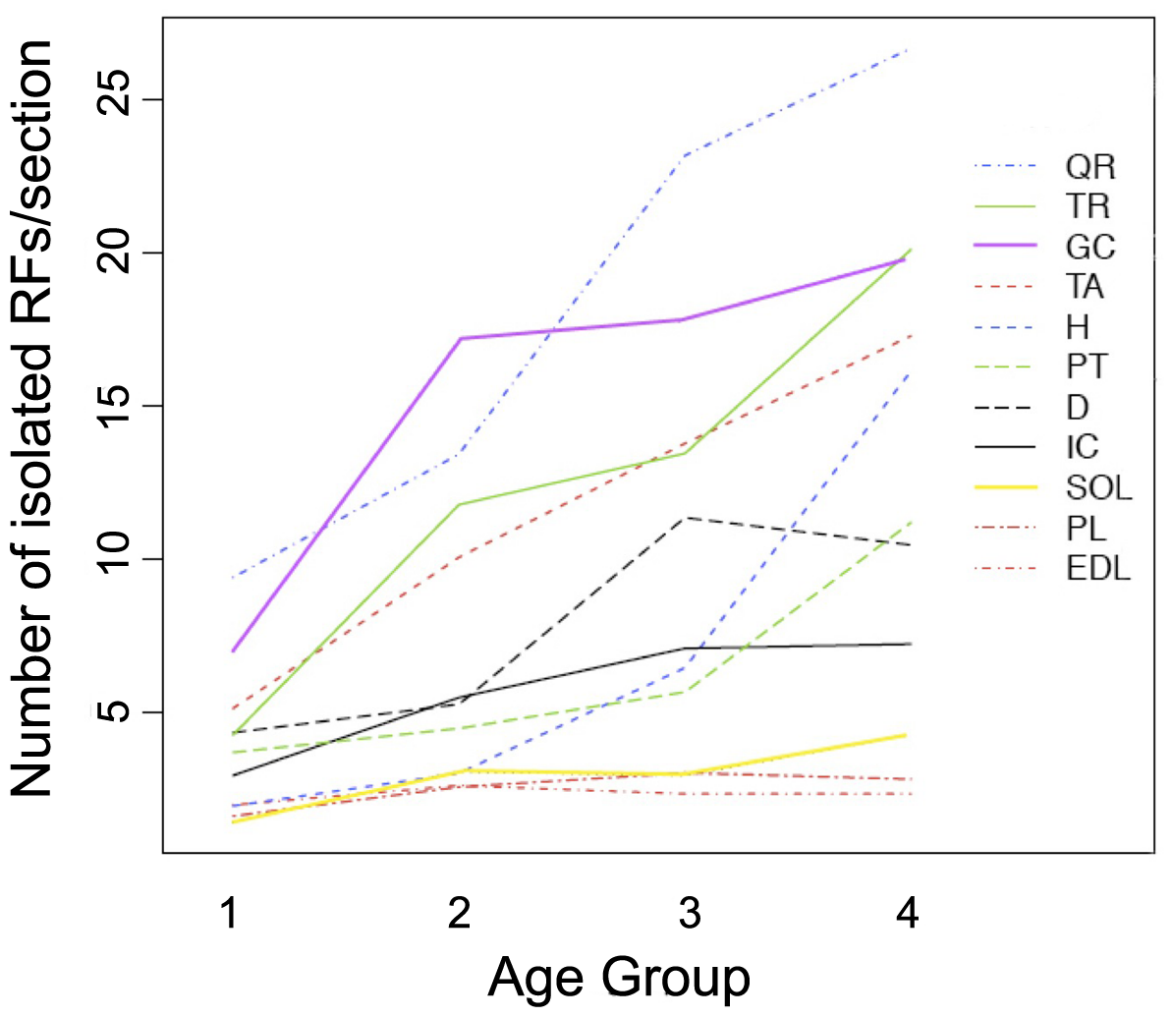
Interaction between the number of isolated RFs and the age of the animals. Chart is organized as in Figure 1B; for each muscle, values were obtained by averaging the absolute number of single dystrophin-positive fibers (i.e., separated by at least two negative fibers from neighboring RFs) found in each section.

As shown in Figure 3, when comparing the different age groups two distinct trends could be seen. For some muscles (EDL, plantaris, soleus and intercostal), the average number of isolated fibers per section remained essentially constant at all time points, whereas for all other muscles there was a distinct increase with ageing. In particular, the difference between groups 1 and 2 was significant (p<0.001) in tibialis anterior, gastrocnemius and triceps; between groups 1 and 3 in tibialis, gastrocnemius, quadriceps, diaphragm and triceps; between groups 2 and 3 in quadriceps and diaphragm; between groups 2 and 4 in tibialis anterior, quadriceps, diaphragm, chest and triceps. Finally, the difference between groups 1 and 4 was significant in all muscles except EDL, soleus and plantaris.

Of extreme interest were the data from cardiac muscle, in which there was a significant increase in the number of isolated RFs between groups 1 and 3 (p<0.05), groups 2 and 4 (p<0.01), groups 3 and 4 (p<0.01) and between groups 1 and 4 (p<0.01).

Lastly, we analyzed how the clusters’ size varied over time in the different muscles. At first this approach did not show any clear trend, likely due to the high variability of the data, which included clusters ranging from two to a maximum of 61 dystrophin-positive fibers (Table 2). We therefore decided to re-analyze our dataset by categorizing the clusters in four classes, based on their size: “small clusters” consisting of 2–4 RF, “medium clusters” with 5–8 RF, “large clusters” with 9–17 RF and “very large cluster” with more than 17 RF. When considering the average number of clusters per section, we found that the values tended to increase with the age of the animals for all cluster sizes but even in the oldest animals small clusters were by far the most common occurrence (e.g., the gastrocnemius of group 4 mice had an average of 10 small clusters and 1.5 medium clusters per section).

**Table 2:** Maximum number of RFs/mm2 per cluster, at each age group.

	Group 1	Group 2	Group 3	Group 4
**TA**	12	15	20	24
**EDL**	8	7	8	11
**SOL**	4	20	14	29
**PL**	3	9	13	22
**GC**	11	21	35	26
**Q**	17	36	23	30
**TRIC**	17	39	27	60
**PT**	13	23	17	17
**D**	8	21	21	19
**IC**	15	10	22	58
**H**	10	8	7	11

Interestingly, cardiac muscle had virtually no medium, large and very large clusters, but the average number of small clusters per section showed a significant increase (p<0.05) between groups 1–4 (0.6 versus 2.8) and groups 2–4 (0.5 versus 2.8).

## Discussion

First described more than 20 years ago [10] [10], the biology of RFs remains to date only partially understood. At present it is hypothesized that dystrophin re-expression is due to epigenetic events that involve the splicing process; these would lead to the non-incorporation of multiple exons, which in turn would restore of the open reading frame of the shortened transcript [15]. Such events are thought to take place in individual satellite cells and then be transmitted to their myoblasts progeny [18]. Reversion phenomena present high inter- and intra-individual variability in terms of what areas of the transcript are affected, both in humans [9] and in *mdx* mice [16]; yet, given that they result in a correctly localized dystrophin, all of them must produce a protein with near-intact N-terminus and C-terminus [3], [16]. Such requirement is demonstrated by the complete absence of revertant fibers in a particular *mdx* strain, *Dmd^mdxßgeo^*, in which a ßgal cassette inserted in exon 63 of the dystrophin gene replaces the cysteine-rich and the C-terminal domain [20]. The actual mechanism(s) leading to the splicing alteration are still unknown, though; for example, at present there is no explanation as for why point mutations in the rod-domain (the central, “disposable” region of dystrophin) can be associated with large differences in the number of RF. This is for example the case for *mdx* and *mdx^cv2^* strains (characterized by point mutations in exon 23 and intron 42, respectively), whose muscles display about ten times more revertant fibers than *mdx^cv4^* (point mutation in exon 53) and *mdx^cv5^* mice (point mutations in exon 10) [14].

In 2006, Yokota and colleagues confirmed that the number of RFs was proportional to the age of the animal and that such increase was linked to proliferation of satellite cells, as it was abolished in the absence of regenerative processes [18]. In our experience, we had found that *mdx* mice showed of a high degree of variability both in the number and organization of the RF, even between the same muscles of age-matched individuals (LV and SP, unpublished observation and Table S1). Consequently, in order to have the sample numbers necessary to carry out robust statistical analysis, we decided to include ten animals per age group. We felt that this was an absolute necessity because in all previous reports the power of statistical analyses was limited by the small numbers of animals/muscles.

We considered the following parameters: number of RFs per mm^2^ (something that had never been reported before), the percentage of clustered RFs over the total RFs and the organization/distribution of the RFs, expressed in terms of number of isolated RFs and size of RFs clusters.

When considering the number of revertant fibers per mm^2^, we found a significant increase with the animals’ age, thereby confirming previous observations. However, our data also indicated that the frequency of RFs was not constant amongst different muscles; in particular, diaphragm and pectoralis had a lower incidence than all other muscles. Such finding could be due to a different incidence of the reversion phenomena and/or to a decreased persistence of RFs in these muscles. The first explanation could be linked to intrinsic differences in the satellite cell populations of diaphragm and pectoralis compared to limb muscles. Indeed, satellite cell heterogeneity has been shown in many instances [21] and it is known that satellite cells derived from the diaphragm and ventral trunk muscles express high levels of Pax3, whereas those from limb muscles do not [21], [22].

On the other hand, the lower RFs incidence could depend from a decreased protective effect of dystrophin re-expression, possibly as a consequence of the type of workload and/or the anatomical structure of diaphragm and pectoralis. This hypothesis, however, does not agree with the fact both muscles still exhibit an increase in the percentage of clustered fibers with age.

Tibialis anterior and soleus, on the other hand, showed the highest number of RF/mm^2^. Given that the former is one of the least compromised muscles in *mdx* mice [23], [24], whereas the diaphragm exhibits the most severe dystrophic phenotype –both histologically and functionally– [25], one could wonder about a possible relationship between number of RFs and severity of dystrophic phenotype. Soleus muscle, however, presents a histopathological picture not as severe as diaphragm but more pronounced than tibialis anterior [24], and no functional or histological data are available for pectoralis, the other muscle with the least number of RFs/mm^2^. At present, therefore, there are no sufficient data to establish a definite relationship between number of RFs and dystrophic phenotype in *mdx* muscles. In this regard, we carried out preliminary analyses of macrophages’ distribution in tibialis anterior sections from group 4 mice; our initial data indicate that medium and large RF clusters appear to contain less inflammatory infiltrate compared to RF-free areas (data not shown).

In all analyzed muscles, the number of revertant fibers as a function of time showed an increasing trend but the differences between adjacent age groups were rarely statistically significant, suggesting that such increase was gradual and rather constant during all phases of the animals’ life.

The results obtained from the analysis of the spatial organization of RFs in muscle sections were in agreement the hypothesis of clonal expansion, as the percentage of clustered RF tended to increase with age in all skeletal muscles, as previously reported. However, the number of seemingly newly formed single RF, with the notable exception of soleus, EDL and plantaris, also appeared to increase in time, thereby suggesting that the biological process(es) behind the formation of RFs might not be limited to the first phases of animals’ life. It should be noticed, however, that even though our choice of counting a RF as “single” when separated by at least two dystrophin-negative fibers from the nearest revertant agrees with what has been reported about the mobility of satellite cells *in vivo* ([15] and references therein), there is also the possibility that these apparently newly formed fibers actually derived from the migration of pre-existing revertant satellite cells. Specific experiments with panels of antibodies against different dystrophin epitopes will therefore be necessary to confirm our observation.

To the best of our knowledge, we are the first to report a detailed analysis of the reversion phenomenon in dystrophic heart. At first, the low total number of revertant cardiomyocytes did not allow to establish statistically significant differences between the four age groups in terms of total number of dystrophin-positive cells. In particular, hearts showed an increasing trend with age in the number of RFs/mm^2^ and in the percentage of clustered RFs/section, but neither data reached statistical significance. On the other hand, we found a significant increase with age in the number of isolated RFs/sections and in the number of small clusters. Our results therefore suggest that also in the case of cardiac muscle the formation of new dystrophin-positive cardiomyocytes might parallel the clonal expansion of already formed revertant cells. The data reported here hence suggest the presence of lifelong cardiac regenerative activity; interestingly, the increase in isolated revertant cardiomyocytes is significant not only between distant age groups (which suggests a slow regenerative effect whose effect becomes evident only in the long run) but also between group 3 and 4, thereby suggesting a relatively faster rate of reversion in aged mice. Altogether, these findings might support the hypothesis, so far put forward for normal heart [26], [27], that *mdx* cardiac muscle contains a population of stem cells capable of cardiomyocyte turnover.

The presence of RFs complicates the evaluation of any therapeutic approach for dystrophin restoration, both in animal and human studies. Specifically, even though the number of dystrophin-positive fibers in untreated muscles is generally low, the inter-individual variability can be quite significant, especially in the presence of factors that affect muscle regeneration. This in turn can affect the readout of experiments in which the effectiveness of the treatment is not particularly high. Besides, in time course experiments that consider animals at different ages the confounding effect of RFs could be even more detrimental [9], [14]. Our data represent the first comprehensive reference for those who use *mdx* mice as an experimental model for treating DMD; in fact, having normalized our data as number of RFs/mm^2^ will provide a useful tool for evaluating the outcome of dystrophin-restoration experiments. Last but not least, having analyzed many different muscles can also provide useful indications on which muscles to choose for the evaluation of a therapeutic approach in experimental animals of different age ranges.

## Supporting Information

Figure S1 **Example of dystrophin immunostaining in different muscles.** Panel A, *wild type* tibialis anterior from a 18-month old mouse. Panel B, a single revertant fiber in a group 3 soleus muscle; panel C, clusters of various sizes in a group 4 tibialis anterior; panel D, a very large cluster in a group 4 triceps; panel E, two revertant cardiomyocytes from a group 3 heart. Nuclei are stained in blue with DAPI; overlays were obtained from two images of the same field, using Photoshop CS3. Scale bar (100 µm) applies to all panels.
(TIF)

Table S1 **Minimum and maximum number of RFs/mm^2^ in different muscles, at each age group.**
(DOCX)
