# Supplementary figures and images for: Revertant Fibers in the mdx Murine Model of Duchenne Muscular Dystrophy: An Age- and Muscle-Related Reappraisal
Source: PLoS One. 2013 Aug 28;8(8):e72147. doi: 10.1371/journal.pone.0072147 (PMC3756063; doi:10.1371/journal.pone.0072147)

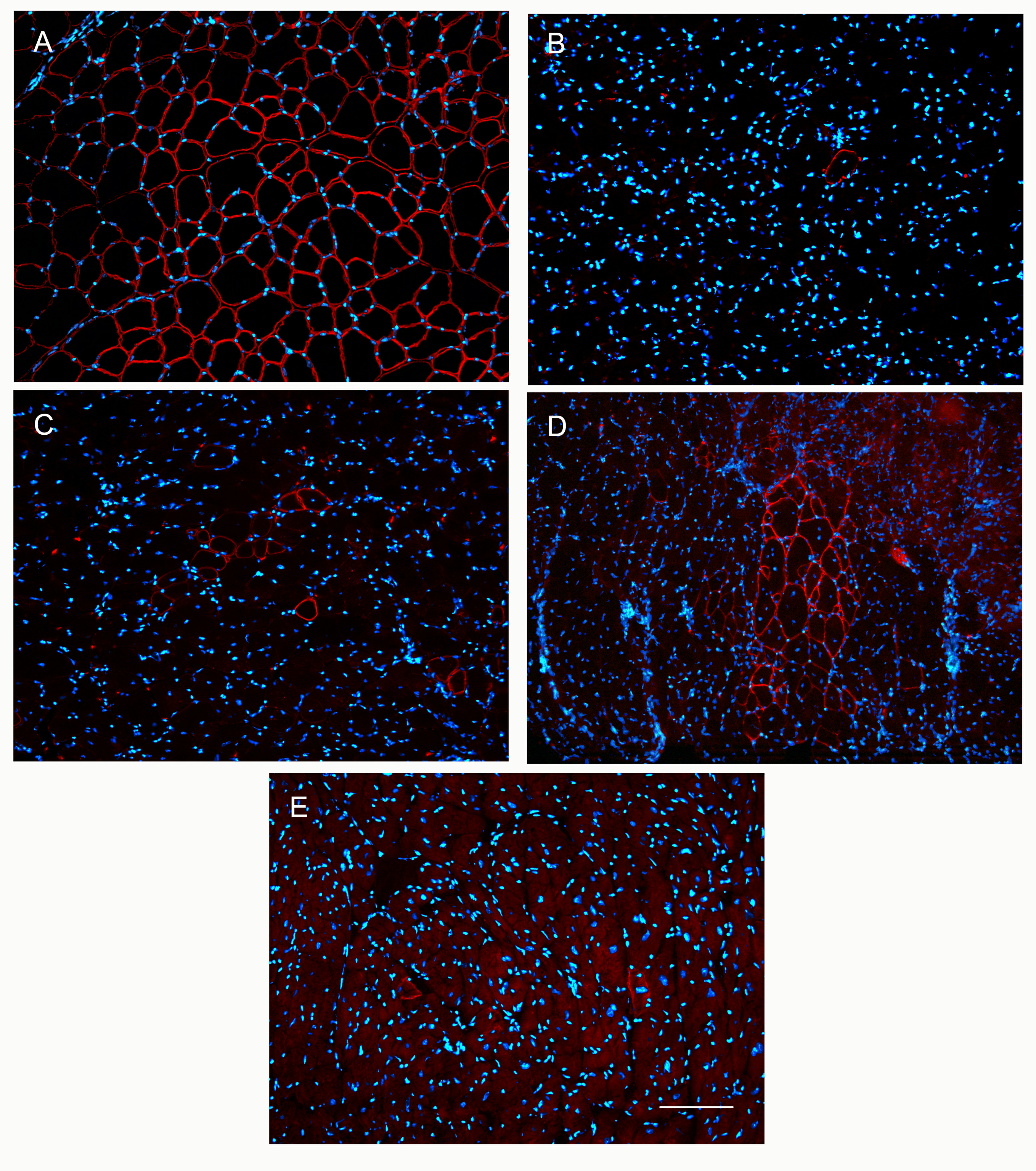

Supplement: Figure S1 — Example of dystrophin immunostaining in different muscles. Panel A, wild type tibialis anterior from a 18-month old mouse. Panel B, a single revertant fiber in a group 3 soleus muscle; panel C, clusters of various sizes in a group 4 tibialis anterior; panel D, a very large cluster in a group 4 triceps; panel E, two revertant cardiomyocytes from a group 3 heart. Nuclei are stained in blue with DAPI; overlays were obtained from two images of the same field, using Photoshop CS3. Scale bar (100 µm) applies to all panels. (TIF) [file pone.0072147.s001.tif]
