# Supplementary material for: Revertant Fibers in the mdx Murine Model of Duchenne Muscular Dystrophy: An Age- and Muscle-Related Reappraisal
Source: PLoS One. 2013 Aug 28;8(8):e72147. doi: 10.1371/journal.pone.0072147 (PMC3756063; doi:10.1371/journal.pone.0072147)
Supplement: Table S1 — Minimum and maximum number of RFs/mm2 in different muscles, at each age group. (DOCX) [file pone.0072147.s002.docx]

**Table S1: Minimum and maximum number of RFs/mm^2^ in different muscles, at each age group**

|  | **GROUP 1** | **GROUP 2** | **GROUP 3** | **GROUP 4** |
| --- | --- | --- | --- | --- |
| **TA** | 0; 13.81 | 0.44; 11.89 | 0.83; 12.47 | 1.65; 17.39 |
| **EDL** | 0; 17.3 | 0; 11.34 | 0; 12.72 | 0; 30.15 |
| **SOL** | 0; 9.23 | 0; 9.52 | 0; 26.52 | 0; 30.37 |
| **GC** | 0; 5.4 | 0.55; 11.07 | 0; 10.75 | 0.33; 13.08 |
| **PL** | 0; 10.81 | 0; 22.5 | 0; 16.34 | 0; 16.67 |
| **Q** | 0.19; 2.63 | 0.39; 5.43 | 0.88; 8.79 | 1.45; 20 |
| **TRIC** | 0; 12.43 | 0.29; 6.49 | 0.9; 8.96 | 0.52; 27.3 |
| **PT** | 0; 12.42 | 0; 12.87 | 0; 8.38 | 0; 9.82 |
| **D** | 0; 3.98 | 0; 11.44 | 0; 7.38 | 0; 13.69 |
| **IC** | 0; 4 | 0; 4.51 | 0; 22.42 | 0; 27.91 |
| **H** | 0: 2.91 | 0; 1.28 | 0; 2.2 | 0; 3.74 |

Values are referred to the single muscle sections that had the lowest and highest number of RFs, considering all the animals in each group.
